# Skin of Color Representation for Atopic Dermatitis on TikTok: Cross-Sectional Analysis

**DOI:** 10.2196/48635

**Published:** 2023-10-27

**Authors:** Alyssa Abdelnour, Nicholas Comeau, Kurt Ashack

**Affiliations:** 1 College of Human Medicine Michigan State University Grand Rapids, MI United States; 2 Dermatology Associates of West Michigan Grand Rapids, MI United States

**Keywords:** dermatology, dermatologist, derm, derma, telederm, teledermatology, skin, color, atopic, dermatitis, eczema, TikTok, media, atopic dermatitis, disparity, social media, post, clinical presentation, educational information, digital platform, digital health

## Introduction

Atopic dermatitis affects millions of people worldwide, with potentially substantial impacts. With the popular social media platform TikTok, dermatologists can share skin health information with a larger audience. Since launching in 2016, TikTok has had over 1 billion monthly active users and is the world’s fastest-growing social media platform [1]. The quality of dermatology-related content on TikTok has been shown to be largely inaccurate, with patients, rather than board-certified dermatologists, being the most popular creators [1,2]. Additionally, the quality and inclusivity of information on these platforms may be limited, particularly for patients with skin of color (SoC) [3]. Despite increasing efforts to represent patients with Fitzgerald skin types III-VI in dermatologic literature, additional evidence is needed [4]. We aim to evaluate the quality of dermatology content and representation of patients with SoC on TikTok. We hope to identify opportunities to improve the dissemination of accurate and inclusive information.

## Methods

TikTok was searched with the term #eczema on July 27, 2022, and the first 136 videos were assessed. We included nonduplicative English videos with relevant content. Of the 136 videos, 119 met the inclusion criteria and 17 did not. Videos by board-certified physicians were categorized as physician; those by individuals without recognized medical qualifications were categorized as nonphysician.

Two independent researchers rated 119 videos using the DISCERN criteria, assessing the information quality and reliability [5]. DISCERN is a well-established tool with criteria designed to assess the quality of health-related information and materials, evaluating various aspects of the content, including information accuracy and source reliability. Cohen κ was calculated at 0.9375, indicating high interrater reliability and agreement in the DISCERN analysis.

A single researcher analyzed videos for SoC representation, assessing examples of patients affected by atopic dermatitis using the Fitzpatrick scale when included. Accurately assessing Fitzpatrick skin type based on videos may have limitations.

## Results

Of the 119 videos, 102 were created by nonphysicians and 17 by physicians (dermatologists: n=14; nondermatologists: n=3). The average DISCERN score was 1.26 (Table 1) for nonphysician posts and 2.24 for physician posts (*t*_236_=0.0006496729527; *P*<.001). Of the nonphysician posts, 30 contained images or videos featuring SoC. Only 1 of the physician videos and none of the dermatologist videos contained SoC. Nonphysician posts had three times more views than physician posts. There was no significant difference (*P*=.21) between the number of views of physician and nonphysician videos. Among physicians, dermatologists had higher average view counts compared to nondermatologists (597,357 vs 105,033).

**Table 1:** DISCERN scores for skin of color (SoC) inclusion in TikTok posts created by nonphysicians and physicians.

	Videos, n	Views, mean (SD)	DISCERN score, mean (SD)	Videos with SoC, n (%)
Nonphysician	102	1,711,392 (2,553,537)	1.25888 (0.2694)	30 (29.4)
Physician	17	510,476 (605,589)	2.24265 (0.4377)	1 (5.9)
Dermatologist	14	597,357 (636,551)	2.21205 (0.3891)	0 (0.0)
Nondermatologist	3	105,033 (26,626)	2.38542 (0.2818)	1 (33.3)
Total	119	1,539,832 (2,400,033)	1.39942 (0.4607)	31 (26.1)

## Discussion

Dermatologists can use the visual nature of their field to engage a broad audience on social media platforms like TikTok [6]. By creating informative videos that showcase diverse clinical presentations and treatment options, dermatologists can enhance the dissemination of accurate and educational health content.

Our study underscores the need for high-quality health content and improved SoC representation in atopic dermatitis–related posts on TikTok. We observed a significant difference in the DISCERN scores between physician and nonphysician content (*P*<.001), suggesting that physicians can potentially elevate the quality and reliability of health information on social media platforms.

This study also highlights a gap in SoC representation in posts about atopic dermatitis. Physicians appear to lag behind nonphysicians in this regard, most likely due to the available number of physician videos compared to nonphysician videos. Individuals with SoC often manifest with clinical findings that look different than patients with a lighter skin tone. Therefore, by improving SoC representation in TikTok videos, such as including atopic dermatitis examples of patients with Fitzpatrick skin types III-VI, we can bridge this informational gap and ensure a more diverse audience receives accurate and relevant content. Inclusivity in dermatological education is not only a matter of equity but also a means to enhance the overall impact and reach of health-related messages on TikTok. To address this, health care professionals should better represent the diverse patient population they serve on social media platforms. The low viewership of physician videos on TikTok suggests that even if physicians improved SoC representation in their videos, the impact may be limited.

Another limitation was our relatively small sample size, comprising 119 videos. Although we believe our sample was representative of a general search result for atopic dermatitis, other TikTok videos meeting our inclusion criteria were possibly not captured. Furthermore, we exclusively searched TikTok, so the generalizability of our findings to other social media platforms or online resources may be limited.

Our study underscores the current underrepresentation of SoC in atopic dermatitis content on TikTok while highlighting the potential for dermatologists and health care professionals to enhance accessibility and accuracy by leveraging social media, emphasizing the importance of an expanded and reliable online presence by physicians.

## Abbreviations

SoC: skin of color
